# Depression in the elderly and dementia with Lewy bodies: A case report of a challenging diagnosis

**DOI:** 10.1192/j.eurpsy.2024.1102

**Published:** 2024-08-27

**Authors:** E. Smaoui, D. Mnif, N. Reguaieg, F. Guermazi, S. Sakka, I. Baati, J. Masmoudi

**Affiliations:** ^1^Neurology department, CHU Habib Bourguiba; ^2^Psychiatry department, CHU Hédi Chaker, Sfax, Tunisia

## Abstract

**Introduction:**

Depression and dementia with Lewy bodies (DLB) are two fairly common pathologies in the elderly which can have similar presentations or be associated and therefore pose a diagnostic challenge.

**Objectives:**

We propose to illustrate, through our case, the diagnostic and therapeutic challenge of these two pathologies.

**Methods:**

We present the case of Ms. S. BA aged 67, without organic or psychiatric history, admitted to the psychiatry department for massive anxiety and insomnia. The troubles date back to nineteen months when the patient isolated herself, remained bedridden, lost her appetite and no longer slept. The evolution quickly led to the appearance of an excessive agitation. The patient became distracted, talking and laughing to herself, and ran away from the house. She consulted several free-lance psychiatrists and received several antipsychotic medications without improvement. The admission interview revealed a very anxious patient with a difficult contact. Her speech was centred on well-detailed visual hallucinations with themes of death. The neurological examination was difficult at first. She was started on haloperidol and clonazepam. After 2 days, neurological examination showed a parkinsonian syndrome and a temporal disorientation. Other cognitive functions were difficult to assess. The two diagnoses evoked were DLB and a characterized depressive episode with psychotic features. Standard workup showed mild anaemia and thrombocytopenia. Brain MRI and electroencephalogram and immune tests were normal. However, PET imaging was not available in our hospital. Haloperidol was immediately stopped and the patient was treated with an anticholinergic corrector in combination with quetiapine at 200 mg. The evolution was characterized by a significant reduction in anxiety and visual hallucinations with a marked improvement of the parkinsonian syndrome. Depressive symptoms took the forefront of the clinical presentation; hence we associated sertraline with quetiapine. The subsequent evolution showed a clear improvement in the depressive symptoms with total resolution of the parkinsonian symptoms and a normal cognitive evaluation.

**Results:**

In our case, the clinical evolution constituted a key element in the diagnostic orientation. So far, it is unlikely that our patient has DLB and the diagnosis retained was a characterized depressive episode with psychotic and melancholic features. Depression in the elderly can have atypical presentations, and raise the possibly of other differential diagnoses. Diagnostic uncertainty should not delay the implementation of treatment.

**Conclusions:**

The choice of molecules must take into account the associated somatic symptoms for a better tolerance. In the absence of a biological or iconographic examination with good sensitivity and specificity, the therapeutic test remains the only way to decide.

**Disclosure of Interest:**

None Declared

